# N- and C-Terminal Domains of the Calcium Binding Protein EhCaBP1 of the Parasite *Entamoeba histolytica* Display Distinct Functions

**DOI:** 10.1371/journal.pone.0005269

**Published:** 2009-04-22

**Authors:** Ruchi Jain, Shivesh Kumar, Samudrala Gourinath, Sudha Bhattacharya, Alok Bhattacharya

**Affiliations:** 1 School of Life Sciences, Jawaharlal Nehru University, New Delhi, India; 2 School of Environmental Sciences, Jawaharlal Nehru University, New Delhi, India; INSERM U567, Institut Cochin, France

## Abstract

*Entamoeba histolytica*, a protozoan parasite, is the causative agent of amoebiasis, and calcium signaling is thought to be involved in amoebic pathogenesis. EhCaBP1, a Ca^2+^ binding protein of *E. histolytica*, is essential for parasite growth. High resolution crystal structure of EhCaBP1 suggested an unusual arrangement of the EF-hand domains in the N-terminal part of the structure, while C-terminal part of the protein was not traced. The structure revealed a trimer with amino terminal domains of the three molecules interacting in a head-to-tail manner forming an assembled domain at the interface with EF1 and EF2 motifs of different molecules coming close to each other. In order to understand the specific roles of the two domains of EhCaBP1, the molecule was divided into two halves, and each half was separately expressed. The domains were characterized with respect to their structure, as well as specific functional features, such as ability to activate kinase and bind actin. The domains were also expressed in *E. histolytica* cells along with green fluorescent protein. The results suggest that the N-terminal domain retains some of the properties, such as localization in phagocytic cups and activation of kinase. Crystal structure of EhCaBP1 with Phenylalanine revealed that the assembled domains, which are similar to Calmodulin N-terminal domain, bind to Phenylalanine revealing the binding mode to the target proteins. The C-terminal domain did not show any of the activities tested. However, over-expression in amebic cells led to a dominant negative phenotype. The results suggest that the two domains of EhCaBP1 are functionally and structurally different from each other. Both the domains are required for structural stability and full range of functional diversity.

## Introduction

Calcium (Ca^2+^) is a ubiquitous intracellular signal responsible for controlling numerous cellular processes in wide spectrum of organisms. Cells respond to an extra-cellular stimulus by a transient change in intracellular Ca^2+^ concentration ([Ca^2+^]_i_) which, in turn, is sensed by calcium binding proteins (CaBPs) [1]. Ca^2+^ signaling also plays a vital role in the biology of many protozoa including *Entamoeba histolytica* [2]. *E. histolytica* genome encodes a large repertoire of CaBPs as revealed by a motif-based search for EF-hand containing proteins suggesting an extensive Ca^2+^-based signaling network in this organism [3]. Many of these proteins are expressed in proliferating trophozoites suggesting that these are likely to be functional proteins [3, Padhan unpublished observations].

Our laboratory previously identified a 14.7 kDa calcium binding protein, EhCaBP1 [4], from *E. histolytica*. This protein shares 29% sequence identity with the ubiquitous CaBP, Calmodulin (CaM). However, this protein is functionally distinct from CaM [5]. EhCaBP1 is an essential protein, as down regulation of its expression blocks proliferation of the parasite [6]. A phagocytosis deficient *E. histolytica* mutant, L6, showed reduced expression of EhCaBP1, further confirming its involvement in phagocytosis [7]. Detailed analysis showed the involvement of EhCaBP1 in different forms of endocytosis, such as pinocytosis and erythrophagocytosis [8]. EhCaBP1 is likely to participate in the initiation step of endocytosis as it associated transiently with phagocytic cups and was not found in phagosomes [9]. Interestingly, the recruitment of EhCaBP1 to the phagocytic cups was not dependent on its ability to bind Ca^2+^. The mechanism by which EhCaBP1 is recruited to the phagocytic cups is not yet clear, although its ability to bind both F- and G-actin directly has been demonstrated [8].

Crystal structure of EhCaBP1 showed an unusual arrangement of the domains of EhCaBP1 [10]. The region connecting EF hands I and II was found to be less flexible with extended conformation. On the other hand, the two glycines (G63, G67) present in the central linker region makes it more flexible as compared to CaM. The N-terminal domains of three molecules of EhCaBP1 interact in a head to tail manner to form a trimer. In the trimeric form, hydrophobic pockets are formed at each interface, and inter-pocket distance is almost equal to the distance between the hydrophobic pockets in the extended structure of CaM. Hence, it is highly plausible that both the domains carry distinct functional properties thus conferring several/ additional functional features to the protein. Moreover, CaM and CaM-like proteins (ex: Troponin C, Myosin ELC's) bind to their respective target proteins by anchoring to the hydrophobic residues. Particularly, CaM binds to different types of target binding motifs, where the hydrophobic residues are separated by 1–10, 1–14 and 1–16 residues [11].

In the present study, we decided to decipher the roles of the two domains of EhCaBP1 and to understand the binding mode of EhCaBP1 to its targets.

## Results

### Expression and characterization of recombinant domains

**Figure 1:**
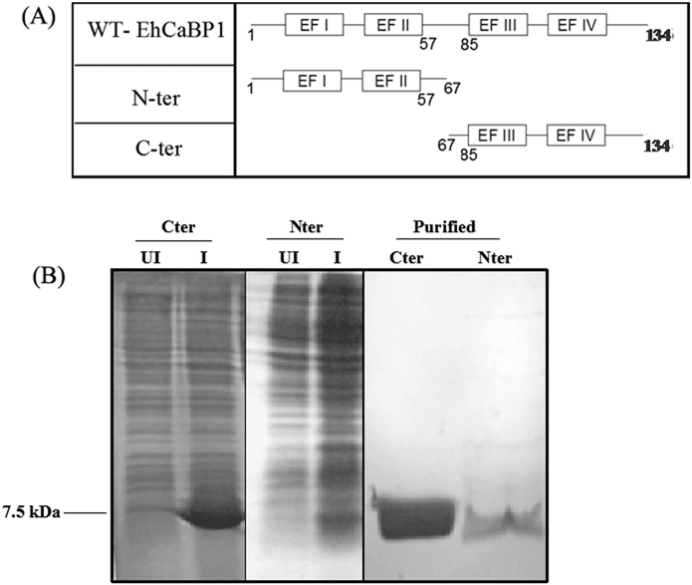
Cloning and Expression of EhCaBP1 domains. (A) Schematic representation of EhCaBP1 domains. Nter protein lacks the carboxy terminus of the protein and contained only the initial 66 amino acids, while Cter protein lacks the initial 66 amino acids. (B) The induction and purification profiles for both the Nter and Cter domains are shown. Induced lysates from the bacterial cells expressing the EhCaBP1 domains are resolved on a 15% SDS-PAGE and the gel is stained with Coomassie Brilliant Blue R-250.

The nucleotide sequences encoding the two domains were separately cloned in *Escherichia coli* expression vector pET 3(c) as described in “materials and methods”. The amino terminal domain (Nter) contained amino acids 1–66 and the carboxy terminal domain (Cter) contained amino acids 67–134 (Figure 1A). The integrity of each construct was checked by nucleotide sequencing. The domains were expressed in presence of the inducer IPTG and the expressed proteins were analysed by SDS-gel electrophoresis (Figure 1B). Purification of the expressed proteins from *E. coli* was carried out essentially as described before [4]. The results show that the Cter domain is expressed at a higher level compared to the Nter domain. At higher concentrations, the domains were found to be less soluble compared to the whole protein (data not shown here).

**Figure 2:**
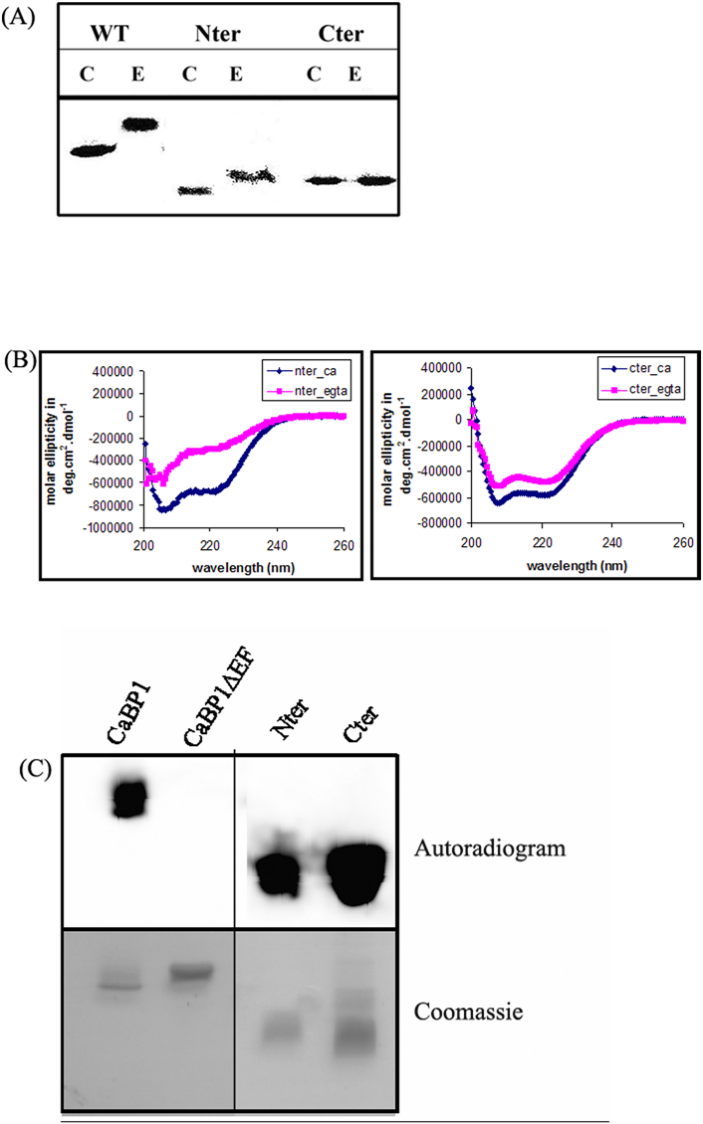
Characterization of Ca^2+^ binding property of the EhCaBP1 domains. (A) Gel mobility shift of Ca^2+^-bound and Ca^2+^- free forms of EhCaBP1 (WT) and respective domains. Recombinant proteins are purified from *E. coli* lysates expressing EhCaBP1 WT or indicated domains. Five micrograms of purified proteins (as indicated) are subjected to electrophoresis on a 15% SDS-PAGE in presence of 5 mM Ca^2+^ (C) or 2 mM EGTA (E). The proteins are stained with Coomassie Brilliant Blue R-250. (B) Far UV spectra of EhCaBP1 domains in presence and absence of Ca^2+^ are shown separately. (C) Ca^2+^ binding by the domains is also checked by ^45^Ca overlay assay. The figure represents an autoradiogram (upper panel) and the corresponding SDS-PAGE gel is stained with Coomassie Brilliant Blue R-250. CaBP1ΔEF, a Ca^2+^ insensitive mutant of EhCaBP1 [9] was used as a negative control. The vertical line indicates that few unwanted lanes have been deleted from the gel picture.

The Ca^2+^ binding ability of a protein can be checked by a number of methods. The methods, such as mobility shift assay and circular dichroism spectroscopy (CD) measures changes in the conformation of the protein after binding Ca^2+^ and therefore are indirect approaches for determining Ca^2+^ binding. The Nter and Cter domains were subjected to mobility shift assay where conformation change on binding Ca^2+^ was visualized on a SDS-PAGE gel. The Ca^2+^ bound form of the Nter domain underwent a mobility shift similar to that observed for the full-length protein. No significant shift was observed in the case of Cter domain (Figure 2A). This may be due to a small conformational change undetectable by SDS-PAGE. CD spectroscopy was subsequently performed to decipher any subtle conformation change on Ca^2+^ binding (Figure 2B). It is evident from the spectra that both Nter and Cter domains underwent conformational changes in presence of Ca^2+^. As expected Nter showed a larger degree of change in helicity compared to the Cter domain (10% as compared to 3% in Cter). The ability of both the domains to bind Ca^2+^ was confirmed by a direct ^45^Ca^2+^ binding assay where western blotted proteins are incubated with radioactive ^45^Ca^2+^ (Figure 2C). EhCaBP1ΔEF, a mutant form that does not bind Ca^2+^ was used as a negative control [9]. The results clearly showed that both the domains bound Ca^2+^. It appears from these results that though both the domains bind Ca^2+^, the consequence of binding is not the same. Nter domain undergoes a major conformation change whereas the change is much less for Cter. In this respect, Nter domain behaved like the whole EhCaBP1.

### Functional characterization of the domains

**Figure 3:**
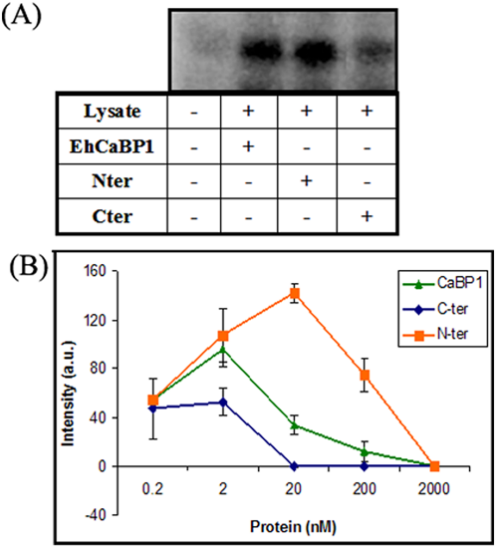
Ability of EhCaBP1 domains to activate endogenous kinase(s). (A) *E. histolytica* cell-free lysate (25 µg) is used as the source of kinase and histone type III (15 µg) as the substrate. EhCaBP1, Nter or Cter (2 nM) is added to the reaction mixture at varying concentration and the assay is performed using [γ-^32^P] ATP as the phosphate donor. The concentration of Ca^2+^ used is 10 µM. The reaction products are separated on a 12% SDS-PAGE, air dried and autoradiography is done. The first lane is histone alone. (B) The amount of radioactivity incorporated into histone as determined by densitometry. The reaction mixtures are precipitated and the incorporated counts are measured using liquid scintillation counter. The graph represents the intensity measurement of three independent experiments ±s.d.

EhCaBP1 is known to activate endogenous kinase(s) in a Ca^2+^ dependent manner [5]. The ability of the domains to activate these kinase(s) was tested as described before, using histone phosphorylation visualized by autoradiography [9] (Figure 3A). While Nter could activate the endogenous kinase more efficiently than the complete EhCaBP1, Cter showed a marked reduction in activity of about 50–60% of the control (Figure 3B).

**Figure 4:**
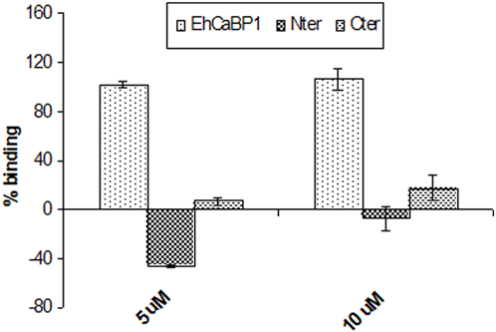
Actin binding properties of EhCaBP1 domains. The ability of the domains to bind G-actin is tested using solid phase assay at different concentrations (5 and 10 µM). The histogram shows the relative mean intensity ±s.d. of three independent experiments.

Since EhCaBP1 has been shown to bind G actin directly each domain was also tested separately for their ability to bind G-actin using a solid phase assay [8]. The results showed no significant binding for either of the domains, suggesting that G-actin binding requires intact protein (Figure 4).

### Expression of individual domains in transfected *E. histolytica* trophozoites

**Figure 5:**
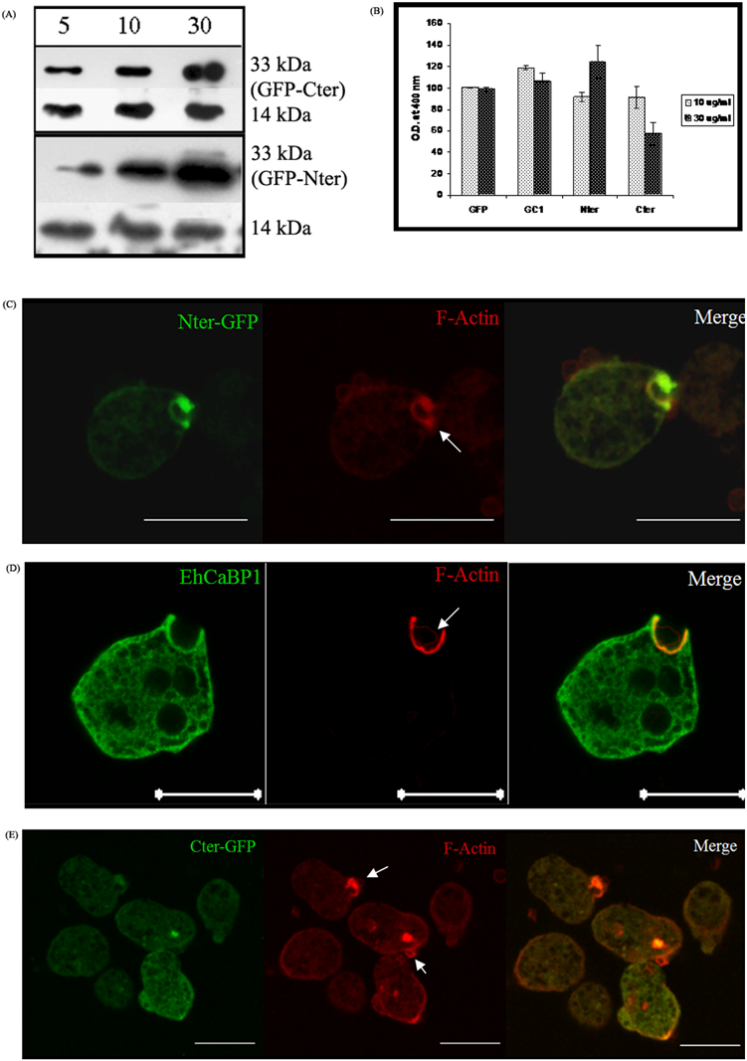
Expression and localization of GFP tagged EhCaBP1 domains in *Entamoeba* trophozoites. (A) Immunoblot analysis of the lysates prepared from cells over-expressing GFP tagged Cter or Nter domains of EhCaBP1grown at 5, 10, 30 µg/ml G418. Fifty microgram of whole cell lysate is resolved on a 12% SDS-PAGE and blotted on to a nitrocellulose membrane. The blots were probed with anti-EhCaBP1 (1∶2000) followed by anti-Rabbit HRPO (1∶10000) and visualized using chemiluminescence substrate. (B) Erythrophagocytosis in cells over-expressing EhCaBP1 domains. The cells expressing Nter-GFP, Cter-GFP, GFP-EhCaBP1 (GC1) or vector alone (GFP) are grown at 10 µg/ml and 30 µg/ml of G418. Erythrophagocytosis is measured after incubating 10^5^ amoebae with 10^7^ RBC for 20 min at 37°C. The histogram shows relative mean optical density ±s.d of three independent experiments. (C–E) Nter (C), WT HM1∶IMSS (D) and Cter (E) was visualized during erythrophagocytosis by immunolocalization in cells expressing Nter-GFP (C) or Cter-GFP (E) at 30 µg/ml G418. EhCaBP1 domains are stained with anti-GFP antibody (green) and phalloidin (red) and viewed using CSLM. Full length EhCaBP1 (D) is stained with a polyclonal antibody against EhCaBP1. Arrows indicate position of some of the RBCs. Scale bars represents 20 µm.

The domains were expressed in *E. histolytica* cells in order to study their function. The DNA fragments encoding the two domains were separately cloned in the *Entamoeba* shuttle vector, pEh-NEO-GFP as described in “Materials and Methods”. These constructs were then transfected in *E. histolytica* cells, generating over-expressing Nter-GFP and Cter-GFP cell lines. The expression of the fusion protein was checked by immunoblotting, using anti-EhCaBP1 antibody (Figure 5A). Densitometric analysis of the immunoblot showed a 3.5 fold increase in the expression of Nter-GFP and 2.5 fold in case of Cter-GFP at 30 µg/ml of G418 as compared to the cells maintained at 5 µg/ml of the antibiotic.

It has been shown that EhCaBP1 has a crucial role in the initiation of erythrophagocytosis and erythrophagocytosis has been linked to the pathogenesis in amebiasis [12]. In order to find out the level of erythrophagocytosis in Nter-GFP and Cter-GFP cell lines these cells were incubated with RBCs (Figure 5B). There was no significant difference in the level of erythrophagocytosis between Nter-GFP cells and the cells containing the vector containing GFP alone. However, a marked reduction (40%) was seen in case of Cter-GFP cells when grown at 30 µg/ml of G418. This suggests that over-expression of Cter domain results in a dominant negative phenotype with respect to erythrophagocytosis. Absence of dominant negative effect in Nter-GFP cells suggests that this domain is likely to behave like the full length EhCaBP1 protein. Over expression of full length EhCaBP1 also did not change significantly the level of erythrophagocytosis [8].

Fluorescence microscopy was used for subcellular localization of expressed domains in amoebic cells during erythrophagocytosis. Arrows in the figures indicate positions of some RBCs. Confocal sections showed the presence of the Nter domain at the phagocytic cup and its complete co-localization with F-actin (Figure 5C). Furthermore, majority of Nter molecules were found around the phagocytic cups and not much in cytoplasm (Figure 5C). The full-length EhCaBP1 protein molecules are found around phagocytic cups as well as in the cytoplasm (Figure 5D). Distribution of Cter protein was quite different. Most of the molecules were found in the cytoplasm, with no specific relation with F-actin at the site of attachment of RBC (Figure 5E). Moreover, in the majority of cells, RBCs were seen bound to the surface and only a few phagocytic cups were observed.

### Structural analysis

**Figure 6:**
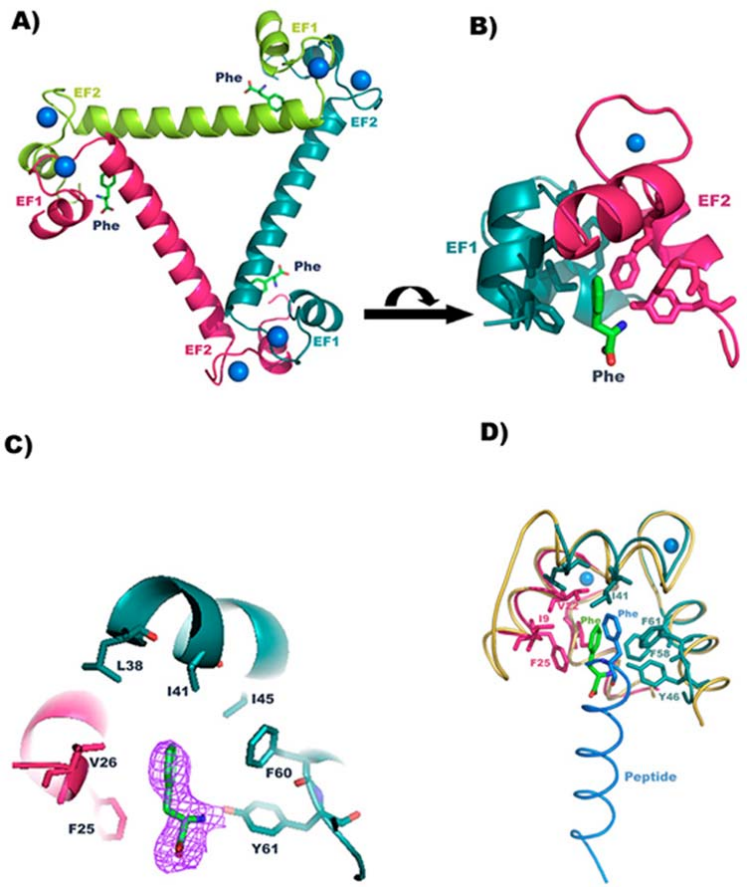
Structural Characterization of EhCaBP1 bound to Phenylalanine molecule. (A)Structure of trimeric EhCaBP1 showing the phenylalanine molecule bound in the hydrophobic pocket formed by the assembled domain. Three molecules interact with each other by head-tail manner forming an assembled domain at the interface. (B) Close view of assembled domain showing phenylalanine in the hydrophobic pocket surrounded by several hydrophobic residues. (C) 2Fo-Fc electron density map of phenylalanine at 2σ level. (D) The N-terminal domain of CaM-peptide complex is superposed on the assembled domain of EhCaBP1-phenylalanine complex. The hydrophobic hankering residue of the peptide in CaM-peptide complex is bound to similar location as Phe in the assembled domain of EF1 of one molecule (hot pink) and EF2 of neighboring molecule (deep teal).

The two EF hand motifs belonging to N-terminal domain of EhCaBP1 are separated by long helix. In contrast, the corresponding EF motifs in CaM are connected by a short loop, thus bringing these two EF hand motifs into close proximity and forming a two EF-hand domain. The N-terminal domain of three molecules of EhCaBP1 participates in domain swapping to form trimers (Figure 6A) [10]. This allows the EF1-hand motif of one molecule to interact with EF2 of an adjacent molecule to form a two EF-hand domain. This assembled domain is similar to that of the two EF hand domains of CaM and TnC. This is essentially facilitated by a couple of critical residues in the linker that separate EF1 and EF2 motifs in comparison to CaM and ELC's [10]. CaM and CaM-like proteins bind to their targets by anchoring hydrophobic residue of the target. Two EF hand motifs of each domain of CaM bind to one hydrophobic residue of the target. To understand the target binding mode, EhCaBP1 was co-crystallized with Phenylalanine (Phe). Crystal structure of EhCaBP1 with Phe showed that the hydrophobic pocket formed at the interface between EF1 and EF2 in the assembled domain is bound to Phe (Figure 6B) with good electron density (Figure 6C). The Phe forms several hydrophobic interactions with Ile 8, Phe 24, Val 21 and Val25 residues of EF hand motif 1 of one molecule and Tyr 61, Phe 60, Phe 57, Ile 40 and Leu 37 residues of EF hand motif 2 of another molecule (Figure 6C).

The Phe-bound assembled domain was superimposed on the N-terminal domain of CaM, bound to hydrophobic IQ motif of cardiac Ca^2+^ channel [13] (Figure 6D). Both structures superimposed very well with an RMS deviation of 1.23 Å. The hydrophobic residue Phe of the peptide (bound to CaM N-terminal domain) and Phe bound to EhCaBP1 assembled domain are located at similar regions. Both ligands bound their targets at their respective hydrophobic pockets.

The distance between the two assembled domains is approximately the same as the N and C-terminal domains of CaM. But the assembled domain can not change its structure after binding to target as CaM wraps around its target. This rigidity in the trimeric structure may be responsible for differential recognition of the targets.

## Discussion

Ca^2+^ signaling plays an important role in amoebic pathogenesis. Ca^2+^ signal is perceived by a set of proteins known as calcium binding proteins. The *E. histolytica* genome encodes a large repertoire of such CaBPs. One of them, EhCaBP1, has been characterized in our laboratory and shown to be essential for the parasite growth [6]. High resolution crystal structure showed an unusual arrangement, with three molecules of EhCaBP1 interacting in a head to tail manner to form a trimer [10]. This arrangement allows the N-terminal EF-hand motif of one molecule to interact with that of an adjacent molecule to form two EF-hand domains, similar to that seen in CaM and TnC. The data was intriguing and prompted us to carry out structure-function studies of the individual domains of EhCaBP1. The results presented here clearly shows that the N-terminal half of this protein is capable of carrying out some of the functions of the full-length protein, such as localization in phagocytic cups along with actin and activation of endogenous kinase in a Ca^2+^ dependent manner. The observation indicating absence of dominant negative phenotype on over-expression, also supports the view that the N-terminal half of the protein is capable of carrying out some functions of the full molecule. This may be due to expressed N-terminal domains forming trimeric complexes with each other as well as endogenous N-terminal domains of EhCaBP1 molecules. However, not all functions can be carried out by the N-terminal half. For example, it could not bind G-actin. The differential behavior suggests that the two halves of the molecule have different functions. The behavior of Cter cells can also be due to alteration in the property of Cter due to its fusion with GFP, the latter being much larger than the former.

It has been demonstrated that both domains of EhCaBP1 have distinct folding features [14], [15]. This is similar to TnC and CaM where N and C-terminal domains were found to be structurally independent and likely to bind different targets [16]–[18]. TnC interacts with only two proteins, troponin I and troponin T. The N-terminal domain functions as the Ca^2+^-specific regulatory switch, while the C-terminal domain plays mainly the structure stabilizing role [19], [20]. On the other hand, domain independence is the key to high level of versatility of CaM [21]. A genetic screen in *Paramecium* has also revealed that the domains of CaM have separable physiological roles [22]. EhCaBP1 is also thought to be involved in multiple pathways as it binds a variety of target proteins observed by immunoprecipitation and mass-spectrometric studies [5, unpublished observation]. It is likely that these domains function independently contributing to the diversity of functions carried out by EhCaBP1.

Our previous biochemical studies have clearly shown that the calcium binding affinity of the EF3 and EF4 are much higher than that of the EF1 and EF2 [23]. This strongly indicates that only EF1 and EF2 are affected by the Ca^2+^ concentration fluctuations around it and C-terminal domain (EF3 and EF4) should be rigid and may not be influenced by Ca^2+^ concentration changes. This is also evident from the results obtained from crystallization studies presented here that the assembled domain in trimer bind to the hydrophobic amino acid revealing the mode of target binding. Therefore the evolution of the CaBPs, such as EhCaBP1 may have been designed to offer both functional and structural diversity suitable for a pathogen to modulate host-pathogen relationship.

## Materials and Methods

### Strains and culture conditions

*Entamoeba histolytica* strain HM1∶IMSS clone 6 was maintained and grown in TYI-S-33 medium containing 125 µl of 250 U ml^−1^ Benzyl Penicillin and 0.25 mg ml^−1^ Streptomycin per 100 ml of medium. Neomycin (Sigma) was added at 10 µg ml-1 for maintaining transgenic cell lines.

*Escherichia coli* strains BL21 (DE3) and C 41 were maintained in Luria Broth containing 100 µg ml^−1^ ampicillin.

### Cloning of EhCaBP1 domains in pET 3(c) expression vector

The gene fragments corresponding to the two domains (amino and carboxy) of EhCaBP1 protein were cloned in the bacterial expression vector, pET 3(c). The construct having EhCaBP1 gene in pET 3(c) vector was used as a template and a stop codon was introduced by site directed mutagenesis at position 199. The primers used for the mutation were

Primer NF: 5′ CTATGGATCAATTCAATAACAAGATCTTTCTGATG 3′ and
Primer NR: 5′ CATCAGAAAGATCTTGTTATTGAATTGATCCATAG 3′.

The carboxy terminus of EhCaBP1 was amplified using a pair of primers designed to amplify the region 199–405 of the gene. Nde I and BamH I sites were introduced in forward and reverse primer respectively. The primers used were:

Primer CF: 5′ GCGCATATGGGACAAGATCTTTCTGATG 3′ and
Primer CR: 5′ GGGGGATCCGAGTGAAAACTCAAGG 3′.

The constructs were confirmed by nucleotide sequencing. The constructs carrying the amino and carboxy terminus of EhCaBP1 gene in the bacterial expression vector were further transformed in *E. coli* strains C41 or BL21 (DE3) to produce recombinant Nter or Cter protein respectively.

### Generation of cells over-expressing GFP-tagged EhCaBP1 domains

EhCaBP1 cloned in pEhNEO/GFP vector [9] was used as a template and a stop codon was introduced at 199 bp position by site directed mutagenesis using the primers NF and NR. In order to clone the Cter domain of EhCaBP1 in pEhNEO/GFP vector, the region was amplified using primers:

Primer F: 5′ GCGGGATCCGGACAAGATCTTTCTGATG 3′ and
Primer R: 5′ GGGGGATCCGAGTGAAAACTCAAGG 3′.

Both the constructs were confirmed by nucleotide sequencing. These constructs or the one carrying the WT gene were transfected in *E. histolytica* trophozoites by electroporation as described earlier [8].

### Expression and purification of recombinant EhCaBP1 domains from *E. coli*

The purification of the recombinant EhCaBP1 domains was done as described earlier for WT EhCaBP1 [4] except for a few changes. In case of Cter protein, 5 mM CaCl_2_ was used in elution buffer in place of 10 mM CaCl_2_.

For the growth of recombinant Nter protein, Terrific Broth was used instead of Luria Broth due to very less induction in the latter. Briefly, 2% of the primary culture (overnight grown culture) was used as an inoculum for the secondary culture. The culture was induced with 1 mM IPTG for 5–6 h after it attains an O.D of 1 (normally takes 3–4 h) at 37°C. The purification was further followed as done for WT EhCaBP1.

The purified proteins were finally dialyzed against MilliQ and concentrated using Amicon with a cut off of 3 kDa.

### Circular dichroism spectroscopy

CD measurements were performed using a Jasco-815 spectropolarimeter. Each spectrum was measured in the far-UV region (200–260 nm) and was an average of 5 scans. Scans were done at a protein concentration of 33 µM in the buffer containing 50 mM Tris.Cl, pH 7.0 and 100 mM NaCl using a cuvette of path length 1.0 cm in presence of 5 mM CaCl_2_. Percentage helical content was calculated using the method described by Barrow et al. [24].

### Radioactive ^45^Ca overlay assay

The ability of WT or EhCaBP1 domains to bind Ca^2+^ was tested by radioactive Ca^2+^ overlay assay. Briefly, 2 µg of purified protein was run on a SDS-PAGE and blotted to a PVDF or NC membrane. The blot was first washed with 10 mM Imidazole and 2 mM EGTA for 10 min, followed by two washes with chelex treated Milli Q each for 5 min. It was further incubated in Buffer D (10 mM Imidazole pH 6.8, 60 mM KCl, 5 mM MgCl_2_) for 15–20 min at RT and then 1 µCi [^45^Ca] was added to 15 ml of Buffer D and the incubation was continued for another 1 h with constant slow shaking. The blot was then given a brief and gentle wash with chelex treated Milli Q for 2 min, followed by wash with 50% ethanol for 30 s. The blot was finally air dried and exposed for autoradiography.

### In vitro kinase assay

Total *Entamoeba* cell extract was prepared and the activity of EhCaBP1-dependent kinases was estimated as described previously [25]. Varying amounts of either full length or EhCaBP1 domains was added. The gels were dried and exposed to an X-ray film or an imaging plate and densitometry was done.

Alternately, the reactions were by adding 10% TCA and total protein precipitation carried out at 4°C for 45 min. The reaction mixture was spotted onto a GF/C paper and washed with 5% TCA (10 ml) followed by wash with ethanol (5 ml). The filter was then air dried and counts were taken in Cocktail O.

### Phagocytosis of RBC by trophozoites

RBC uptake was monitored spectrophotometrically by estimating the amount of heme present in the trophozoites as described earlier [8]. Samples were measured against a formic acid blank at a wavelength of 400 nm.

### Solid phase assay

The solid phase assay was used to monitor the binding of EhCaBP1 domains to G-actin as described earlier [8]. Briefly, the wells of a 96-well plate were coated with 5 µM G-actin in PBS overnight at 4°C and were blocked with 3% BSA in PBS for an additional 24 h. After washing with PBS-T, EhCaBP1 (positive control) and target proteins (EhCaBP1 domains) were added to the wells in duplicates at varying concentrations. Bound protein was detected with anti-EhCaBP1 antibody followed by HRPO-linked anti-rabbit IgG using the colorimetric substrate TMB (Sigma). The reaction was stopped with 2 N H_2_SO_4_ and absorbance was monitored at 405 nm with a microplate reader (Bio-Rad, USA).

### Immunofluorescence staining

*E. histolytica* cells were stained using different antibodies as described earlier [9]. Antibody dilutions used were: 1∶500, Phalloidin (Sigma, 1 mg/ml in methanol); Anti-GFP; 1∶200, Anti-Rabbit Alexa 488; 1∶300, Anti-Rabbit Alexa Cy3. The preparations were mounted on a glass-slide using DABCO (1,4-diazbicyclo(2,2,2)octane, SIGMA), 10 mg/ml in 80% glycerol. Sealing of the cover-slip edges was done with nail-paint to avoid drying.

### Confocal laser scanning microscopy

Fluorescent samples were examined on LSM 510 confocal laser scanning microscope (CSLM) (Zeiss, Germany) equipped with a 63× objective. Rhodamine-labeled samples were visualized after excitation at 543 nm using He/Ne Laser and Alexa-green labeled samples after excitation at 488 nm using Argon Laser. Pictures were processed using offline version of LSM 510 software, Zeiss.

### Western analysis

For immunodetection, samples were separated on a 12% SDS-PAGE. The gel was then transferred to a nitrocellulose membrane by semidry method and processed using standard methods. The antigens were detected with polyclonal anti-GFP (1∶2000, Molecular probes), polyclonal anti-EhCaBP1 (1∶3000) and with anti-Rabbit HRPO (1∶10 000, Amersham). ECL reagents were used for visualization (Amersham).

### Crystallization of EhCaBP1-Phe complex

The purified protein was concentrated to 30 mg/ml in 50 mM Tris pH 7.5 buffer containing 10 mM CaCl_2_ and 2 mM phenylalanine. This mixture was kept for crystallization similar to native crystallization condition. The complex was crystallized in hanging drops by mixing equal volumes (3 to 5 µl) of the complex with the precipitant solution containing 63 to 65% MPD, 5 mM CaCl_2_ and 50 mM Acetate buffer pH 4. Rod shaped crystals (400×75×75 µM^3^) of EhCaBP1-Phe appeared at 16°C approximately after one week.

### Data collection and processing

The X-ray diffraction experiments were done at 100 K with EhCaBP1-Phe crystals mounted on cryoloops in mother liquor and flash frozen in liquid nitrogen. These crystals diffracted to 2.9 Å with in-house rotating anode generator (Advanced Instrumentation Facility, JNU). They belong to space group P6_3_ (Table 1) with two molecules per asymmetric unit similar to native structure [10]. The data sets were indexed, processed and scaled with Auto-mar program.

**Table 1:** Data-collection and refinement statistics.

Data Set	EhCaBP1-Phenylalanine
Crystallographic data	
X-ray Source	Microstar
Wave length	1.5418
Space group	P6_3_
Unit-cell parameters (Å)	
*a* = 95.201, b = 95.201, c = 64.287	
**Resolution range (Å)**	**50.0 - 2.8**
R_sym_ (%)	6.1(7.33)
Completeness (%)	97.9(86.1)
Total No. of observations	15536
No. of unique observations	8289
Redundancy	8.9 (5.4)
Average *I/s (I)*	51.92 (2.07)
Crystal mosaicity (°)	0.5
**Refinement**	
Resolution (Å)	50 – 2.8
R factor (%)	25.7 (26.4)
Free _R factor (%)	28.5 (28.8)
Mean B factor	93.4
Number of atoms	
Protein/Ca/water /Phe/Acetate	1024/4/42/2/2
**RMS deviations**	
Bonds (Å)	0.009
Bond angles (°)	1.5
Dihedrals angles (°)	18.8
Improper angles (°)	0.73
Cross validated error	0.50

Values in parentheses are for the last resolution shell. Free R factor was calculated with a subset of 7.5% randomly selected reflections.

### Structure determination

The structure was solved by molecular replacement with Phaser program [26] using the native structure of EhCaBP1 (2NXQ) as the search model. The structure was refined to 2.4 Å resolution by iterative model building by the COOT graphics package [27] combined with conjugate-gradient minimization with bulk solvent correction in CNS [28]. The structure looked similar to native EhCaBP1 structure expect large Fo-Fc density at the interface of EF1 and EF2 of the assembled domain to accommodate Phenylalanine. The final model refined well with good electron density and bound Phe (Figure 6D) and crystallographic R_factor_ and R_free_ (Table 1) values that are within the range of average values for structures refined at this given resolution [29]. Despite acceptable refinement statistics, electron density for the C-terminal half of the molecule was absent similar to the native structure. The water molecules, acetate molecules and phenylalanine molecules were added manually where Fo-Fc electron density at ≥3.0 σ contour level and justified by hydrogen bonds or hydrophobic interactions in the final stages of refinement (Table 1).
